# Sangivamycin and its derivatives inhibit Haspin-Histone H3-survivin signaling and induce pancreatic cancer cell death

**DOI:** 10.1038/s41598-019-53223-0

**Published:** 2019-11-12

**Authors:** Ligia I. Bastea, Laeticia M. A. Hollant, Heike R. Döppler, Elizabeth M. Reid, Peter Storz

**Affiliations:** Department of Cancer Biology, Mayo Clinic Comprehensive Cancer Center, Mayo Clinic, Jacksonville, FL 32224 USA

**Keywords:** Biochemistry, Cancer, Cell biology

## Abstract

Current treatment options for patients with pancreatic cancer are suboptimal, resulting in a five year survival rate of about 9%. Difficulties with treatment are due to an immunosuppressive, fibrotic tumor microenvironment that prevents drugs from reaching tumor cells, but also to the limited efficacy of existing FDA-approved chemotherapeutic compounds. We here show that the nucleoside analog Sangivamycin and its closely-related compound Toyocamycin target PDA cell lines, and are significantly more efficient than Gemcitabine. Using KINOMEscan screening, we identified the kinase Haspin, which is overexpressed in PDA cell lines and human PDA samples, as a main target for both compounds. Inhibition of Haspin leads to a decrease in Histone H3 phosphorylation and prevents Histone H3 binding to survivin, thus providing mechanistic insight of how Sangivamycin targets cell proliferation, mitosis and induces apoptotic cell death. In orthotopically implanted tumors in mice, Sangivamycin was efficient in decreasing the growth of established tumors. In summary, we show that Sangivamycin and derivatives can be an efficient new option for treatment of PDA.

## Introduction

Pancreatic ductal adenocarcinoma (PDA), the most common form of pancreatic cancer is predicted to become the second leading cause of cancer deaths in the USA by 2030^1^. PDA usually is detected at a late stage, and despite extensive research to understand the genetic and molecular underpinnings of this disease, novel treatment options are lacking and the 5-year survival rate of patients has only marginally improved over decades^2^. Current treatment options to target pancreatic tumor cells include FOLFIRINOX and Gemcitabine/NAB-paclitaxel. These combinations often can be very toxic because they barely discriminate between malignant and normal cells or tumors can be unresponsive. Therefore, drugs with higher therapeutic index are needed to debulk tumor cells.

While cell cycle aberrations are a hallmark of cancer, agents that target these tumor-specific vulnerabilities so far have not been very successful in the clinic^3^. Haspin (haploid germ cell-specific nuclear protein kinase), also known as Germ Cell-Specific Gene-2 (GSG2) is a nuclear serine/threonine kinase that phosphorylates histone H3 (H3) at threonine residue 3^4^. This phosphorylation, which is performed by Haspin^5^, mediates binding of survivin to histone H3^6^ and promotes the centromeric recruitment of the chromosome passenger complex (CPC), which consists of survivin, borealin, INCENP and Aurora B, during mitosis^7–9^. Depletion of Haspin prevents CPC recruitment to centromeres and normal alignment of chromosomes at metaphase suggesting a role for this kinase during chromosome segregation^6,7,9^. We here show that Haspin is upregulated in pancreatic cancer cell lines and approximately 55% of investigated patient samples.

Sangivamycin and its derivatives are pyrrolo[2,3-d]pyrimidine nucleosides that function as nucleoside analogues, and for example, in Sangivamycin the nitrogen in adenosine at position 7 is replaced by a carbamoyl-substituted carbon. Pyrrolo[2,3-d]pyrimidine nucleosides have been shown to inhibit DNA/RNA synthesis and mediate cell cycle arrest^10,11^ and have been tested for various diseases. For example, Xylotubercidin *in vitro* showed potency and selectivity against infection with herpes simplex virus type 2 (HSV-2)^12^. Sangivamycin showed anti-tumor activity against murine leukemia^13^, and Toyocamycin has been shown to induce a growth inhibition in pancreatic cancer cell lines by inhibiting the unfolded protein response (UPR)^14^.

For pancreatic cancer, we here show that Sangivamycin and its closely-related compound Toyocamycin target PDA cell lines, and are significantly more efficient than Gemcitabine. We identified Haspin as a main target. Inhibition of Haspin prevented Histone H3 binding to survivin, and targets cell proliferation, mitosis and induces apoptotic cell death. In mice, Sangivamycin was efficient in decreasing the growth of established tumors. In summary, we show that Sangivamycin and derivatives can be an efficient new option for treatment of PDA.

## Results

### Sangivamycin and Toyocamycin induce cell death of PDA cell lines

**Figure 1:**
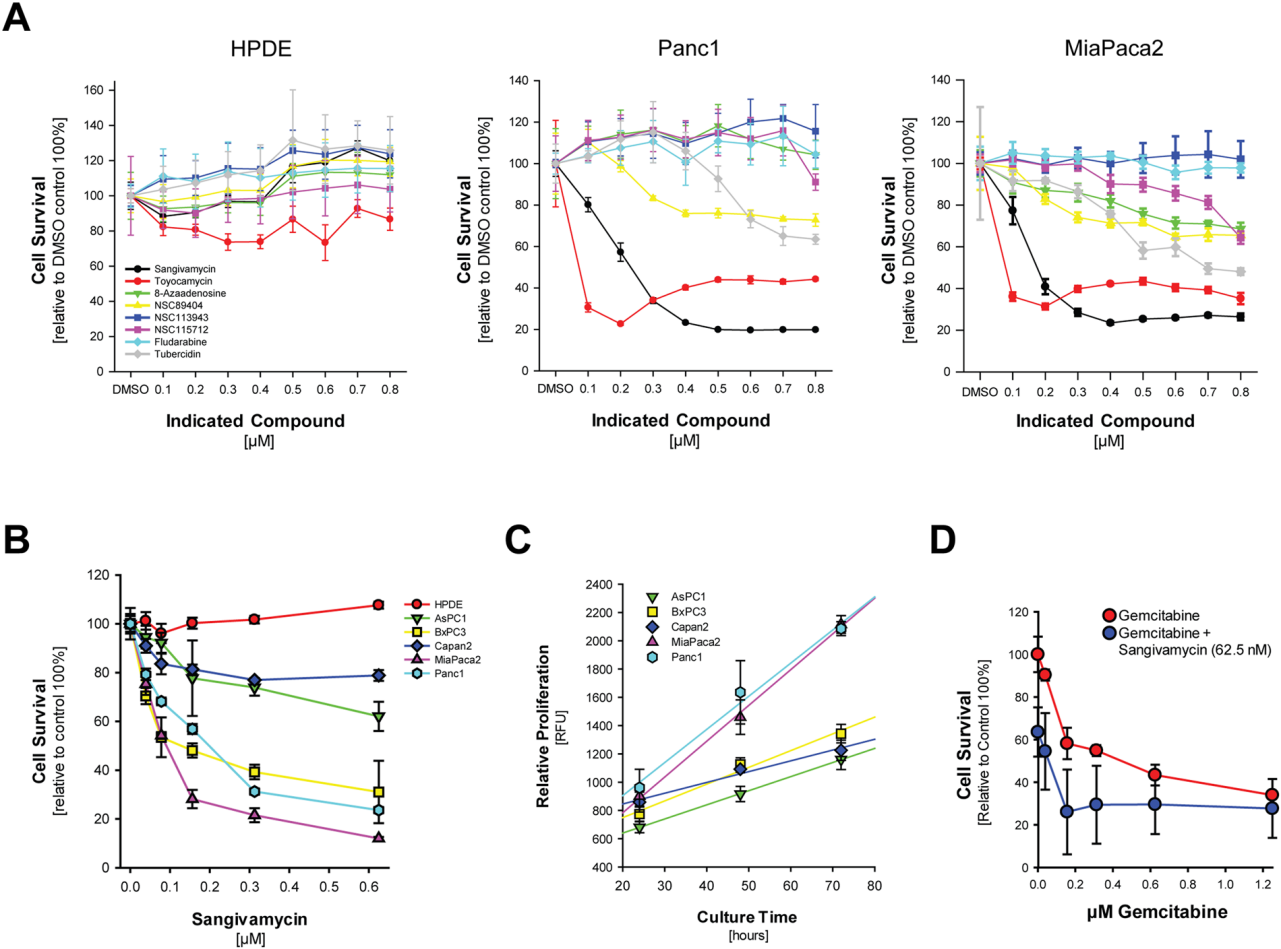
Naturally occurring pyrrolo[2,3‐d]pyrimidine(7‐deazapurine) nucleosides and derivatives efficiently induce human pancreatic cancer cell death. (**A**) Indicated cell lines were seeded in 96 well plates and treated with indicated compounds at indicated dosage for 48 hours. Cell survival was determined using the MTT assay. Error bars represent standard deviation. (**B**) Indicated cell lines were seeded in 96 well plates and treated with Sangivamycin at indicated dosage for 48 hours. Cell survival was determined using the MTT assay. Error bars represent standard deviation. (**C**) Indicated cell lines were seeded in 96 well plates and proliferation was measured after 24, 48 and 72 hours using the CyQUANT Cell Proliferation Assay. Error bars represent standard deviation. (**D**) Panc1 cells were seeded in 96 well plates and treated with Gemcitabine at indicated dosages either in presence of vehicle or Sangivamycin at EC_25_ (62.5 nM) for 48 hours. Cell survival was determined using the MTT assay. Error bars represent standard deviation.

In an effort to identify new drugs that efficiently target pancreatic cancer cells, but also discriminate between normal cells, we analyzed the toxicity of pyrrolo[2,3-d]pyrimidine nucleosides in Panc1 and MiaPaca2. While Sangivamycin and Toyocamycin were most efficient at low dosages, with an EC_50_ of 125 nM in Panc1 and an EC_50_ of 75 nM in MiaPaca2 for Sangivamycin, other pyrrolo[2,3-d]pyrimidine nucleosides showed little or no effects (Fig. 1A). Most importantly, both compounds had no effect on the normal human pancreatic ductal epithelial cell line HPDE (Fig. 1A). While HPDE cells are not responsive to Sangivamycin, a comparison of different pancreas cell lines indicated a variation in sensitivity within lines (Fig. 1B) and this mostly correlated with their relative proliferation rate (Fig. 1C), indicating that pyrrolo[2,3-d]pyrimidine nucleosides target proliferating cells. When compared to treatment with Gemcitabine (Supplemental Fig. S1A), Sangivamycin was more efficient at lower dosage for most pancreatic cancer cell lines. Moreover, treatment of cells with ARC (NSC188491), a compound that had been described to have identical effects as Sangivamycin, also showed strongest effects on highly proliferative cell lines (Supplemental Fig. S1B). A combination of Sangivamycin at EC25 with Gemcitabine at different concentrations indicated that combination of both drugs could even have additive effects (Fig. 1D), and similar results were obtained when ARC (NSC188491) was combined with Gemcitabine (Supplemental Fig. S1C).

### Kinase-targeting spectrum of Sangivamycin and Toyocamycin

**Figure 2:**
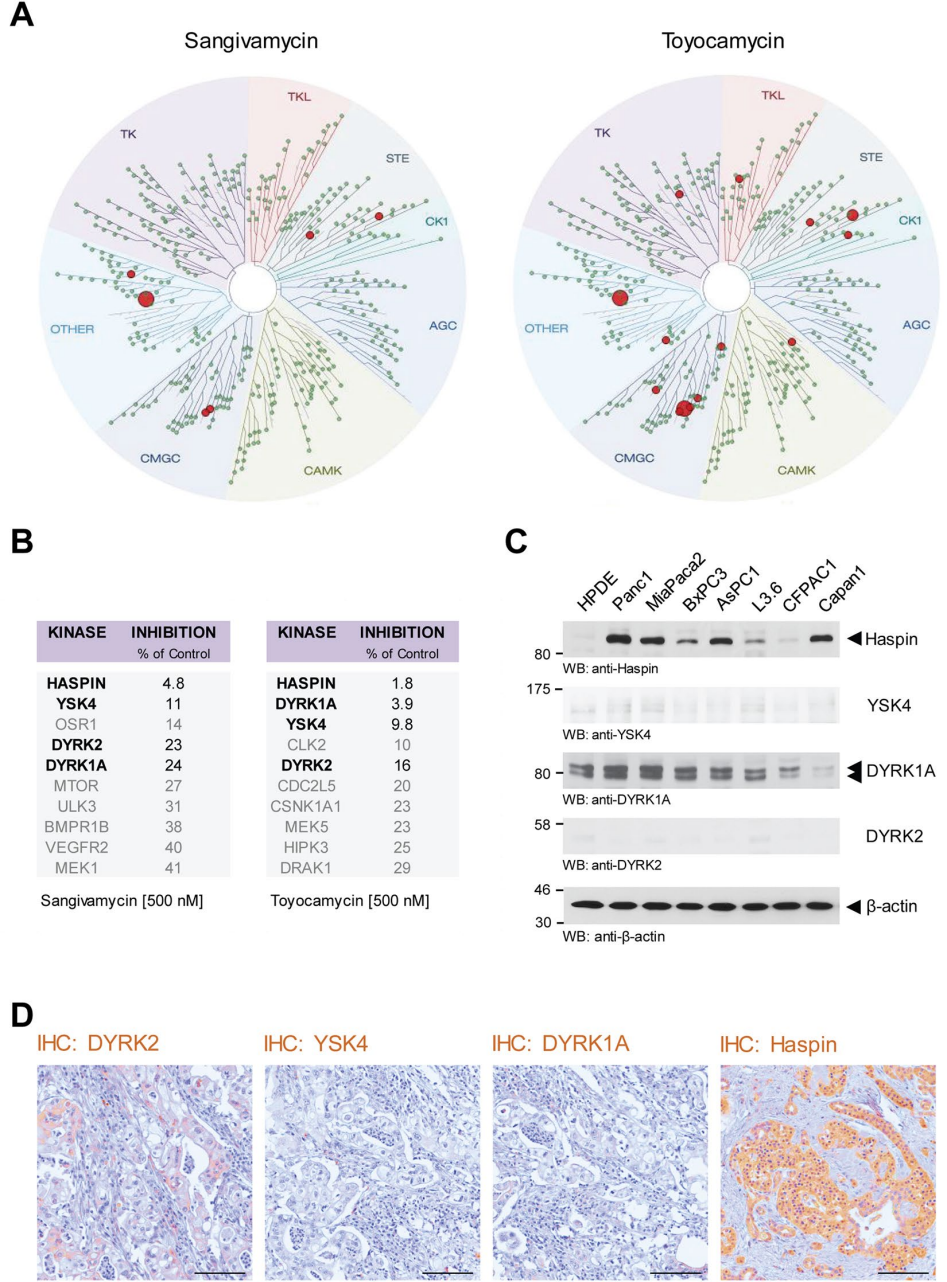
Kinases targeted by Sangivamycin and Toyocamycin. (**A**,**B**) KINOMEscan screening was used to identify the kinase specificity of Sangivamycin and Toyocamycin at a concentration of 500 nM. The top ten hits for each compound is listed in the tables in. (**B**) The four overlapping kinases are highlighted in **bold**. (**C**) Cell lysates of HPDE cells or indicated PDA cell lines were analyzed by Western blot for expression of the main kinases targeted by Sangivamycin and Toyocamycin (anti-Haspin, anti-YSK4, anti-DYRK1A, anti-DYRK2). Staining for β-actin served as control for equal loading. (**D**) Immunohistochemistry (IHC) analysis of human PDA samples using anti-DYRK2, anti-YSK4, anti-DYRK1A, and anti-Haspin antibodies. The bar represents 100 µm.

We next sought to identify the subgroup of kinases that are inhibited by Sangivamycin and Toyocamycin. Therefore we performed a KINOMEscan analysis comprising a set of 456 potential kinase targets (Fig. 2A). Top hit in the S10 group for Sangivamycin (500 nM) was Haspin with a remaining activity of 4.8% of control (100%) and for Toyocamycin also Haspin with a remaining activity of 1.8% of control (100%). Other kinases targeted by both, Sangivamycin and Toyocamycin, were YSK4 (MAP3K19, mitogen-activated protein kinase kinase kinase 19), dual specificity tyrosine kinase 1 A (DYRK1A) and dual specificity tyrosine kinase 2 (DYRK2) (Fig. 2B). Analyses of HPDE cells and a panel of PDA cell lines indicated that YSK4 and DYRK2 are not expressed in pancreatic cancer cell lines (Fig. 2C). DYRK1A is expressed in both normal human pancreatic ductal epithelial cells (HPDE) as well as PDA cell lines. Haspin, however, when compared to HPDE, was increasingly expressed in all PDA cell lines (Fig. 2C). A brief analysis of human PDA tissue samples indicated that these expression patterns in cells may correlate with expression in human cancer, with the exception of DYRK1A, which is not expressed in cancer tissue, but highly upregulated in cultured cell lines (Fig. 2D). We therefore focused in our further studies on Haspin as a potential target for Sangivamycin and Toyocamycin.

### Haspin is increasingly expressed and active in human PDA

**Figure 3:**
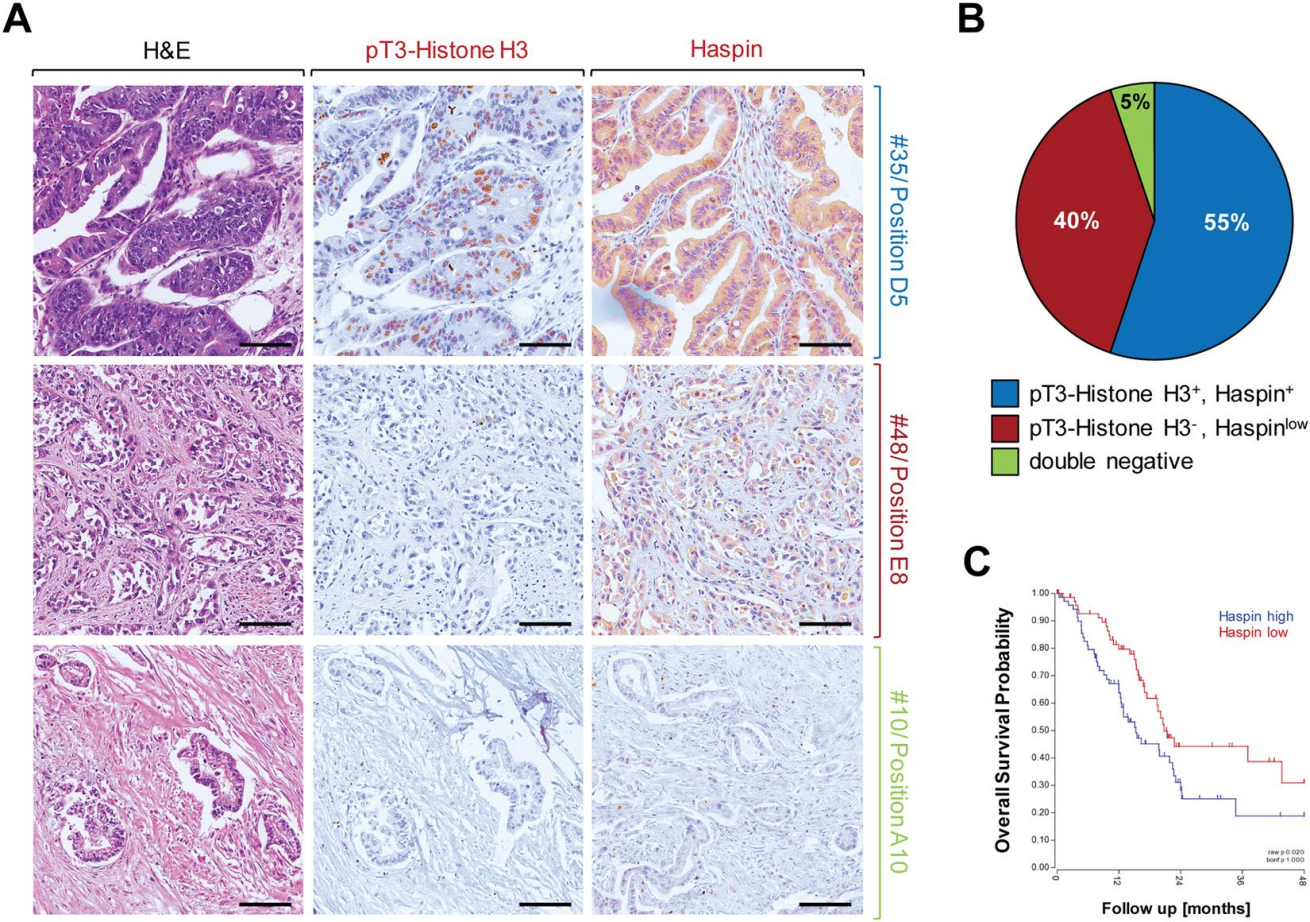
Increased Haspin activity and expression in PDA can be linked to overall survival probability. (**A**,**B**) Tissue microarrays (n = 58 PDA samples) were analyzed by IHC for Haspin or its phosphorylated target Histone H3 using anti-Haspin or anti-pT3-Histone H3 antibodies. (**A**) Shows representative tissues that express Haspin and pT3-Histone H3 (top row), only express Haspin at low level (middle row), or are double negative for both (bottom row). (**B**) Shows a quantification n = 58 samples. (**C**) Overall survival probability of patients either expressing Haspin at high levels (n = 74 samples) or low levels (n = 72 samples). Shown is a Kaplan Curve using TCGA data (GSG_83903; expression cutoff: 29.2540).

We next performed a more detailed analysis of Haspin expression and activity by detecting phosphorylation of its downstream target Histone H3 at threonine residue 3, using n = 60 patient samples. We found Haspin overexpressed and active in approximately 55% of human PDA (Fig. 3A, top row and Fig. 3B). Approximately 40% of samples showed Haspin expression at low levels and no activity towards Histone H3 (Fig. 3A, middle row and Fig. 3B) and 5% of samples were negative for both (Fig. 3A, bottom row and Fig. 3B). Analyses of the overall survival probability of patients indicated that patients with tumors with high Haspin expression levels showed a decrease in survival as compared to patients with tumors that express low levels of this kinase (Fig. 3C).

### Sangivamycin and Toyocamycin inhibit Haspin-Histone H3-survivin signaling

**Figure 4:**
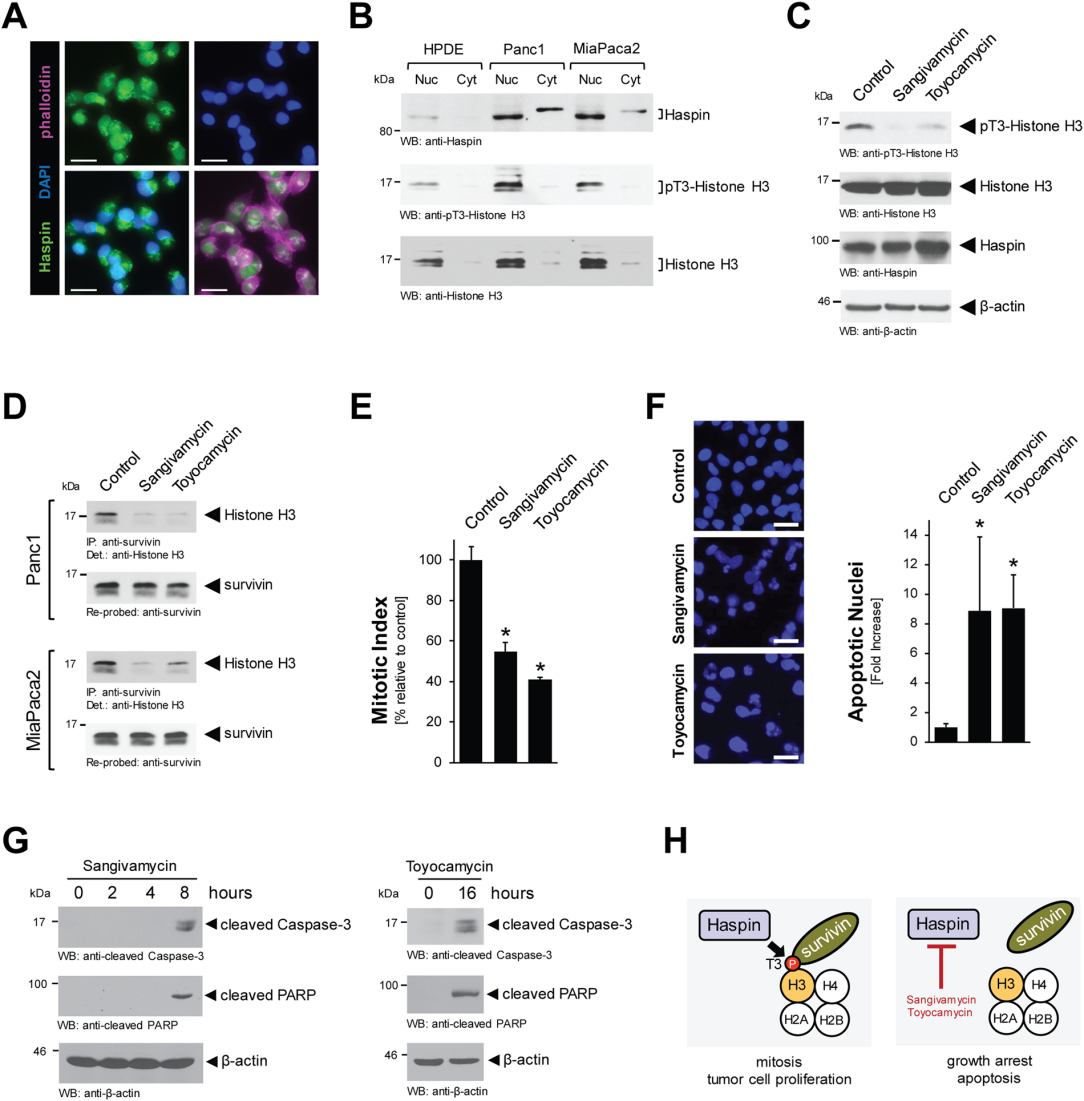
Sangivamycin and Toyocamycin target Haspin-pT3-Histone H3 signaling. (**A**) Localization of endogenous Haspin was determined in Panc1 cell by immunofluorescence with anti-Haspin antibody and co-staining with DAPI (nuclei) and phalloidin (cytoskeletal structures). The bar represents 25 µm. (**B**) Nuclear and cytoplasmic cell fractions were analyzed by Western blot for expression of Haspin (anti-Haspin), phosphorylated Histone H3 (anti-pT3-Histone H3) and total Histone H3. (**C**) Panc1 cells were stimulated for 16 hrs with 500 nM Sangivamycin or Toyocamycin. Whole cell lysates were analyzed by Western blot for expression of Haspin (anti-Haspin), phosphorylated Histone H3 (anti-pT3-Histone H3) and total Histone H3. Staining for β-actin served as control for equal loading. (**D**) Panc1 or MiaPaca2 cells were stimulated for 16 hrs with 500 nM Sangivamycin or Toyocamycin. Survivin was immunoprecipitated and samples were analyzed for co-immunoprecipiated Histone H3. (**E**) Panc1 cells were treated with DMSO (control), Sangivamycin (500 nM) or Toyocamycin (500 nM) for 8 hours and a Mitotic Index assay was performed. (**F**) Panc1 cells were stimulated for 48 hrs with 500 nM Sangivamycin or Toyocamycin as indicated. Left side: Loss of nuclear integrity, indicating apoptosis was determined using DAPI staining. The bar represents 50 µm. Right side: The bar graph shows a quantification of apoptotic nuclei for each condition. The asterisk indicates statistical significance. (**G**) Panc1 cell lysates were treated with Sangivamycin or Toyocamycin for indicated times. Apoptotic signaling was determined by Western blot probing for cleaved Caspase-3 and cleaved PARP. Staining for β-actin served as control for equal loading. (**H**) Proposed mechanism of how Sangivamycin and Toyocamycin target cell proliferation and induce apoptotic cell death.

We next investigated how Sangivamycin and Toyocamycin affect Haspin signaling cascades in pancreatic cancer cells. Immunofluorescence analyses indicated that Haspin, in Panc1 and MiaPaca2 cells, is located at both the nucleus and the cytosol (Fig. 4A and Supplemental Fig. S2A). Overexpression of Haspin and its primarily nuclear localization in PDA cells was confirmed with Western blotting of nuclear and cytosolic extracts. As compared to HPDE cells, both pancreatic cancer cell lines, Panc1 and MiaPaca2, showed increased expression of Haspin in both fractions, as well as increase in phosphorylation of the Haspin target Histone H3 at threonine residue 3 (Fig. 4B). It should be noted that there was a slight difference in the molecular weight of cytosolic and nuclear Haspin, which may be explained by posttranslational modifications.

Histone phosphorylation by Haspin was decreased when cells were treated with Sangivamycin or Toyocamycin (Fig. 4C, for Panc1; Supplemental Fig. S2B, for MiaPaca2). Histone H3 phosphorylation at threonine 3 mediates binding to survivin, and consequently the treatment of cells with Sangivamycin or Toyocamycin abolished this interaction (Fig. 4D). This correlated with an expected decrease in the mitotic index of cells (Fig. 4E). In addition long-term treatment (48 hours) with both compounds increased apoptotic signaling as judged by condensed chromatin and blebbing nuclei seen after DAPI staining (Fig. 4F). Apoptotic effects induced can be seen as early as 8 hours for Sangivamycin and 16 hours for Toyocamycin, as judged by Western blotting for the apoptosis markers cleaved Caspase-3 and cleaved PARP (Fig. 4G). Taken together, this mechanistic data indicates that Haspin overexpression in PDA cells drives mitosis and tumor cell proliferation and that the inhibition of Haspin signaling leads to a disassembly of the H3-survivin protein complex, resulting in growth arrest and eventually apoptosis (Fig. 4H).

### Sangivamycin targets orthotopic PDA tumors *in vivo*

**Figure 5:**
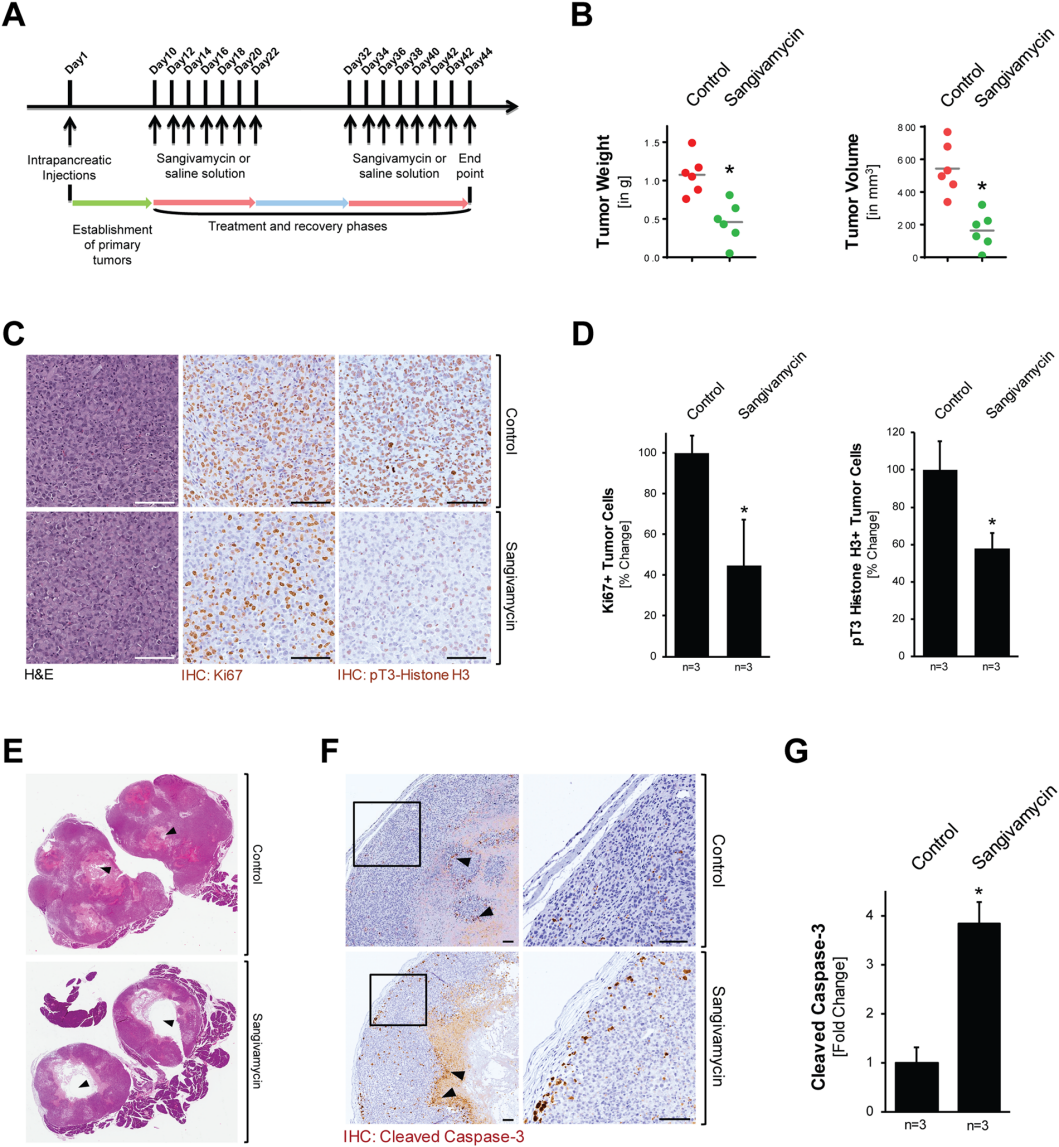
Sangivamycin targets orthotopic PDA tumors *in vivo*. (**A**) Scheme of the timeline of the animal experiment to test the effects of Sangivamycin on orthotopic pancreatic tumors. (**B**) Analyses of tumor weight and tumor volume of mice (n = 6) after indicated treatment. The asterisk indicates statistical significance (p = 0.002 for tumor weight; p = 0.0006 for tumor volume). (**C**) Formalin-fixed tumor samples from mice treated with vehicle or Sangivamycin were analyzed by IHC for expression of either Ki67 or pT3-Histone H3. Shown are representative pictures. The bar represents 100 µm. (**D**) Quantification of IHC staining (n = 3 samples per treatment group; randomly selected) for tumor cells expressing Ki67 and phosphorylated Histone H3. The asterisk indicates statistical significance with p < 0.05. (**E**) H&E staining of tumors shows larger necrosis area (black arrow heads) in Sangivamycin-treated mice, as compared to vehicle-treated mice. (**F**) IHC analysis of control and treated mouse tumors (n = 3 per treatment group) using an anti-cleaved caspase-3 antibody. The bar represents 100 µm. (**G**) Quantification of IHC staining (n = 3 samples per treatment group) for apoptotic tumor cells (cleaved caspase-3 positive tumor cells). The asterisk indicates statistical significance with p < 0.05.

Next we determined if treatment with Sangivamycin can affect tumor growth *in vivo*. Therefore, we orthotopically-implanted Panc1 cells and after establishment of a primary tumor (day 10 after implantation) treated mice with Sangivamycin or vehicle control (n = 6 per treatment group) for indicated time periods (scheme in Fig. 5A). Sangivamycin was administered every other day for 2 weeks with a 10 day recovery break before a second regimen of treatment for 2 weeks. During that time the weight of mice was continuously monitored (Supplemental Fig. S3A). At the end point (day 44) tumors of mice treated with Sangivamycin showed a statistically-significant (approximately 50%) decrease in tumor weight and decrease (approximately 70%) in tumor volume (Fig. 5B). This correlated with an approximately 50% decrease in Ki67 positive cells, indicating inhibitory effects of Sangivamycin on proliferating tumor cells (Fig. 5C and Fig. 5D, left bar graph). No effects on fibrosis within the tumors were observed, suggesting that Sangivamycin does not target fibroblast or macrophages components of the microenvironment (Supplemental Fig. S3B). An approximately 40% decrease in pT3-Histone H3 positive cells in the sangivamycin group indicated that treatment indeed affects Haspin signaling (Fig. 5C,D, right bar graph). In addition to effects on tumor cell proliferation, we observed larger necrotic areas within the primary tumors of Sangivamycin treated mice (Fig. 5E), which may be due to collapsed vasculature structures as determined with anti-CD31 immunohistochemistry (Supplemental Fig. S3C). Moreover, IHC analyses for cleaved caspase-3 indicated a significant (approximately 4-fold) increase in apoptotic cells within the tumor center and in the tumor periphery (Fig. 5F,G).

## Discussion

One caveat in pancreatic cancer treatment is the limited efficacy of existing FDA-approved chemotherapeutic compounds. We here describe the pyrrolo[2,3-d]pyrimidine nucleosides sangivamycin and toyocamycin as potent anti-cancer drugs that efficiently act on cancer cells, either alone or in combination with Gemcitabine (Fig. 1 and Supplemental Fig. S1). *In vivo*, when administered to mice with established orthotopic pancreatic tumors, sangivamycin led to a significant decrease in tumor burden, correlating with a collapse of tumor vasculature, decrease in Ki67 positive cells and an increase in cleaved-caspase 3 expression (Fig. 5 and Supplemental Fig. S3).

Sangivamycin in mice has been shown to remain unmetabolized in spleen and kidney, with a half-life of approximately 50 hours^15^, which indicates that it could be a good candidate for clinical trials. Safety of sangivamycin has been shown in human toxicity study in the 1960s^13^. Sangivamycin so far has not been tested in clinical trials for solid cancers. However, Tubercidin, another pyrrolo[2,3-d]pyrimidine nucleoside which is less efficient in PDA cell lines (Fig. 1A–C), has been tested in phase I clinical studies in patients with various types of advanced neoplastic disease^16^. Of the different tumor types (n = 93) included in this study, only pancreatic tumors showed a response in 3 out of 6 cases. But it should be noted that direct intravenous injections of Tubercidin as performed in this study resulted in local irritation of the veins and nephrotoxicity^16^. Given the nephrotoxic side effects of Tubercidin, similar effects now could be anticipated in clinical trials for PDA, in which Sangivamycin is used alone or in combination therapy. However, our *in vitro* data (Fig. 1) show that the EC_50_ of Sangivamycin or Toyocamycin are significantly lower than the EC_50_ for Tubercidin and other pyrrolo[2,3-d]pyrimidine nucleosides. Therefore, they may be used at a dosage in patients where these adverse effects may not occur.

Kinome scan data implicate Haspin, DYRK1A, DYRK2 and YSK4 as major targets for Sangivamycin and Toyocamycin (Fig. 2A,B). While DYRK2 and YSK4 are not expressed in PDA cell lines and tissue (Fig. 2C,D), DYRK1A is expressed in HPDE control as well as PDA cell lines but was not detected in patient tissue (Fig. 2C,D). Haspin however is increasingly expressed in tumor cell lines as compared to the normal pancreatic ductal cell line HPDE, and also can be detected in patient samples (Fig. 2C,D). So far, a role for Haspin in cancer formation has not been formally established^17^, although Haspin inhibitors such as CHR-6494 have been demonstrated to have antitumor activity in cervical cancer, breast cancer and colon cancer cell lines^18^. A detailed analyses of pancreatic cancer patient samples indicated that Haspin is upregulated in its expression and activity in 55% of analyzed cases of PDA (Fig. 3A,B), and that increased expression of Haspin can be correlated with decreased overall patient survival (Fig. 3C).

Treatment with Sangivamycin and Toyocamycin decreased the mitotic index of cells (Fig. 4E). This may be explained by previously published data showing that inhibition of Haspin by the small molecule inhibitor CHR-6494 causes mitotic catastrophe^18^. Haspin is expressed in the nucleus of proliferating cell lines, where it regulates the alignment of chromosomes in metaphase and phosphorylates Histone H3 at threonine residue 3 (T3) during prophase in mitosis^5^. T3-phosphorylation of Histone H3 facilitates its binding to survivin, which contributes to the formation of the chromosome passenger complex (CPC) during mitosis^7–9^. Sangivamycin and Toyocamycin inhibit Haspin, resulting in decrease of Histone H3 phosphorylation and its interaction with survivin (Fig. 4D). Such dysregulation of signaling in cells can cause a delay in progression through early mitosis, and it was shown for the sangivamycin derivative 5-iodotubericidin that it displaces the CPC from centromeres^19^.

The activity of Haspin in PDA may be regulated through epigenetic changes induced by a downregulation of histone lysine-specific methyltransferase 2D (KMT2D), which acts as a tumor suppressor^20^. KMT2D in PDA cells mediates H3K4me3 methylation^20^, and H3K4me3 strongly decreases Histone H3 substrate recognition by Haspin^21^. The decrease in KMT2D and resulting decrease in H3K4 triple methylation may provide an explanation of how Haspin is activated in PDA to facilitate histone H3 phosphorylation and tumor cell proliferation.

While our data link the inhibition of Haspin to a decrease of mitosis and cell proliferation, we also observed an increase in apoptotic cells after treatment with Sangivamycin or Toyocamycin (Fig. 4G). For ARC (NSC188491), a compound that has identical activity to sangivamycin^22^, it was shown that it induces apoptosis in tumor cells in a p53-independent manner^23^. This suggests that ARC and sangivamycin may be attractive new possibilities for the treatment of PDA tumors with functionally-inactive p53. The apoptotic effects obtained after treatment with Sangivamycin and Toyocamycin may be explained by the dislocation of survivin (Fig. 4E). Survivin is member of the inhibitor of apoptosis (IAP) family which inhibits caspase activation and programmed cell death, and often is overexpressed in human tumors^23^. Indeed, PDA has been shown to express high levels of nuclear survivin, and this has been identified as a marker for poor prognosis^24^.

Taken together, our data indicate that that Sangivamycin and some of its derivatives efficiently-target PDA cell lines. We identified Haspin as a main target and show that its inhibition decreases cell proliferation and induces apoptotic cell death. In mice, Sangivamycin was efficient in decreasing the growth of established tumors, indicating that this compound could be an efficient new option for treatment of PDA.

## Materials and Methods

### Cell lines, antibodies and reagents

HPDE (human pancreatic ductal epithelial) cells (normal control) were obtained from Dr. M-S. Tsao (Ontario Cancer Institute, Ontario, Canada) and maintained as described elsewhere^25^. All pancreatic ductal adenocarcinoma cell lines were from the American Type Culture Collection (ATCC, Manassas, VA) and were maintained as suggested by the ATCC. All PDA cell lines are routinely authenticated via their short tandem repeat (STR) profile (latest verification: May, 2019). Panc1-LUC cells were generated by lentiviral infection with luciferase (lentivirus for luciferase expression described in^26^). Antibodies used for Western blotting, immunohistochemistry and immunofluorescence are described in detail in Supplemental Table 1. Secondary HRP-linked anti-mouse or anti-rabbit antibodies were from Jackson ImmunoResearch (West Grove, PA). DAPI was from Sigma-Aldrich (St. Louis, MO) and phalloidin from Invitrogen (Carlsbad, CA). 2′-Deoxy-2′,2′-difluorocytidine (Gemcitabine, NSC613327) was from Selleckchem (Houston, TX). 4-Amino-7-(β-D-ribofuranosyl)-7H-pyrrolo[2,3-d]pyrimidine-5-carboxamide (Sangivamycin, NSC65346) and 7-(β-D-Ribofuranosyl)-7H-pyrrolo[2,3-d]pyrimidin-4-amine (Tubercidin, NSC56408) were from Sigma. The compounds 4-Amino-6-hydrazino-7-(β-D-ribofuranosyl)-7H-pyrrolo[2,3-d]pyrimidine-5-carboxamide (ARC, NSC188491), 4-Amino-5-cyano-7-(D-ribofuranosyl)-7H-pyrrolo(2,3-d)pyrimidine (Toyocamcyin, NSC63701), 1-(β-D-Ribofuranosyl)-1H-pyrazolo[3,4-d]pyrimidin-4-amine (NSC89404), (4aR,6 R,7 R,7aS)-6-(4-Amino-7H-pyrrolo[2,3-d]pyrimidin-7-yl)tetrahydro-4H-furo[3,2-d][1,3,2]dioxaphosphinine-2,7-diol 2-oxide (NSC115712), 4-Amino-6-bromo-7-(β-D-ribofuranosyl)-7H-pyrrolo[2,3-d]pyrimidine-5-carboxamide (NSC113943), 3-Pentofuranosyl-3H-[1,2,3]triazolo[4,5-d]pyrimidin-7-amine (8-Azaadenosine, NSC72961) and 2-Fluoroadenosine 5′-(dihydrogen phosphate) (NSC312887) were obtained from the NCI/DTP Open Chemical Repository (http://dtp.cancer.gov).

### KINOMEscan analysis

KINOMEscan analyses^27,28^ to determine the kinase specificity of Sangivamycin and Toyocamycin were performed by LeadHunter^TM^ Discovery Services, DiscoveRx Corporation (Fremont, CA).

### Cytotoxicity assays

Cells were seeded in 96 well plates at numbers indicated in the figure legends and treated the next day as indicated. 48 hours after treatment, cell viability was determined using MTT. For MTT assays, 10 µl of MTT in PBS (5 mg/ml) were added per well. Cells were incubated at 37 °C for four hours and then 100 µl solubilization solution (10% SDS in 0.001 M HCl) was added over night. Plates were analyzed by reading at 600 nm using a SynergyHT plate reader (Bio Tek Instruments, Winooski, VT).

### Proliferation assays

Indicated cell lines were seeded in 96 well plates and proliferation was measured after 24, 48 and 72 hours using the CyQUANT Cell Proliferation Assay (Thermo Fisher Scientific, Waltham, MA) according to the manufacturer’s protocol.

### Mitotic index assay

The mitotic index was determined using the chemiluminescent Mitotic Index Assay kit from Active Motif (Carlsbad, CA). Briefly, 20,000 cells per well were seeded in a 96 well white clear bottom plate. The next day, the cells were treated for 8 hours with indicated concentrations of Sangivamycin, Toyocamycin or DMSO control. After fixation, cells were incubated overnight with pS28-Histone H3 monoclonal antibody followed by an HRP-conjugated secondary antibody. Chemiluminescence was read after two minutes incubation with the Chemiluminescent Working solution provided by the kit. The readings were normalized for cell number using Crystal Violet staining.

### Cell Extracts, immunoblotting, immunoprecipitation and PAGE

Nuclear and cytoplasmic cell fractions were prepared as previously described^29^. For total cell extracts, cells were washed twice with ice-cold PBS (140 mM NaCl, 2.7 mM KCl, 8 mM Na_2_HPO_4_, 1.5 mM KH_2_PO_4_, pH 7.2) and lysed with lysis buffer (50 mM Tris-HCl pH 7.4, 1% Triton X-100, 150 mM NaCl, 5 mM EDTA pH 7.4) plus Protease Inhibitor Cocktail (PIC, Sigma-Aldrich). Lysates were vortexed, incubated on ice for 30 minutes, centrifuged (13,000 rpm, 15 minutes, 4 °C), and the supernatant was collected. Supernatants were subjected to SDS-PAGE (Western blotting) or proteins of interest were immunoprecipitated by one hour incubation with specific antibody (2 µg) followed by 30 minutes incubation with Protein G Sepharose^TM^ (GE Healthcare Bio-Sciences AB, Uppsala, Sweden). Immune-complexes were washed three times with TBS (50 mM Tris-HCl pH 7.4, 150 mM NaCl), resolved in 20 µl TBS and 2x Laemmli buffer and subjected to SDS-PAGE.

### Immunofluorescence

Cells were stimulated as indicated, washed 3 times with PBS (5 min, RT) and then fixated with 4% paraformaldehyde (15 min, 37 °C). Following fixation, cells were washed 3 times with PBS (5 min, RT), quenched with 100 mM glycine in PBS (1 min, RT) and permeabilized with 0.1% Triton X-100 in PBS (10 min, RT) or 0.1% SDS (1 min, RT). Following permeabilization, cells were washed 3 times with PBS (5 min, RT) and blocked with 10% NGS and 0.05% Tween 20 in PBS (blocking solution) for 30 min at room temperature. Samples were incubated with indicated primary antibodies (for dilutions see Supplemental Table 1) in 3% BSA and 0.05% Tween 20 in PBS overnight at 4 °C. Following five washes with PBS (5 min, RT), samples were incubated (1 hour, RT in the dark) with secondary antibodies at 1:500 in 3% BSA and 0.05% Tween 20 in PBS, DAPI diluted at 1:1000, or Alexa Fluor 633-phalloidin diluted at 1:40 in PBS (20 min, RT, in the dark). After further washing with PBS, ibidi mounting media was added to the cells in ibidi slide wells. Samples were evaluated using either a IX71 Fluorescent Microscope or an IX81 DSU Spinning Disc Confocal (Olympus, Center Valley, PA).

### Immunohistochemistry

Slides were deparaffinized and rehydrated as previously described^30^. Antigen retrieval was performed in sodium citrate buffer (10 mM, pH 6.0) and tissue samples were treated with 3% H_2_O_2_ (5 min), washed with 0.5% Tween 20/PBS, and blocked with Protein Block Serum-Free Solution (Agilent, Santa Clara, CA; 5 min, RT). Primary antibodies were diluted in Antibody Diluent Background Reducing Solution (Agilent). Specific antibodies used and dilutions are listed in Supplemental Table 1. Staining was visualized using EnVision Plus Anti-Rabbit Labelled Polymer Kit (Agilent), or biotin-streptavidin (Biocare Medical, Concord, CA) 2-step conjugation when primary goat antibodies were used. H&E staining was performed as described previously^30^. ScanScope XT scanner and ImageScope software (Aperio, Vista, CA) were used to capture images.

### Animal experiments

All animal experiments have been approved by the Mayo Clinic Institutional Animal Care and Use Committee (IACUC) under protocol A26309-09. For orthotopic implantation of tumor cells, 6 weeks old athymic nude mice were intrapancreatically-injected with Panc1-LUC cells (250,000 cells) in 30 µl of growth factor-reduced phenol-red free Matrigel (BD Biosciences, San Jose, CA) using a 30 G needle and a glass syringe. Tumor formation was confirmed after 7 days using the IVIS Spectrum Imaging System by IP injection of mice with D-luciferin (150 mg/kg body weight in 0.1 ml PBS). At day 10 after tumor cell implantation, 6 mice per treatment group were treated every 2 days (via IP injection) with 1.6 mg/kg body weight of Sangivamycin diluted in saline with 5%DMSO, 15% Cremophor EL and 5% glucose or solvent solution alone. The treatment schedule is shown in Fig. 5A.

### Human PDA samples

Tissue microarrays (PA805) were purchased from US Biomax, Rockville, MD.

### Quantification and statistical analysis

All cell biological and biochemical experiments have been performed at least 3 times. For animal experiments, if not stated otherwise in the figure legends, pancreatic samples from n = 3 mice have been used for quantification analyses. IHC data was quantified by manual counting of positive cells or by using the Aperio Positive Pixel Count Algorithm. Data are presented as mean ± SD. P values (if not stated otherwise in the figure legends) and were acquired with the unpaired student’s *t*-test with Welch’s correction using Graph Pad software (GraphPad Inc., La Jolla, CA). p < 0.05 was considered statistically significant.

## Supplementary Information

Supplementary Figures and Table

## Data Availability

All data generated or analyzed during this study are included in this published article (and its Supplementary Information files).
