# Supplementary material for: Sangivamycin and its derivatives inhibit Haspin-Histone H3-survivin signaling and induce pancreatic cancer cell death
Source: Sci Rep. 2019 Nov 12;9:16588. doi: 10.1038/s41598-019-53223-0 (PMC6851150; doi:10.1038/s41598-019-53223-0)
Supplement: Supplementary file 1 — Supplementary Figures and Table [file 41598_2019_53223_MOESM1_ESM.pdf]

Supplemental Figure S1

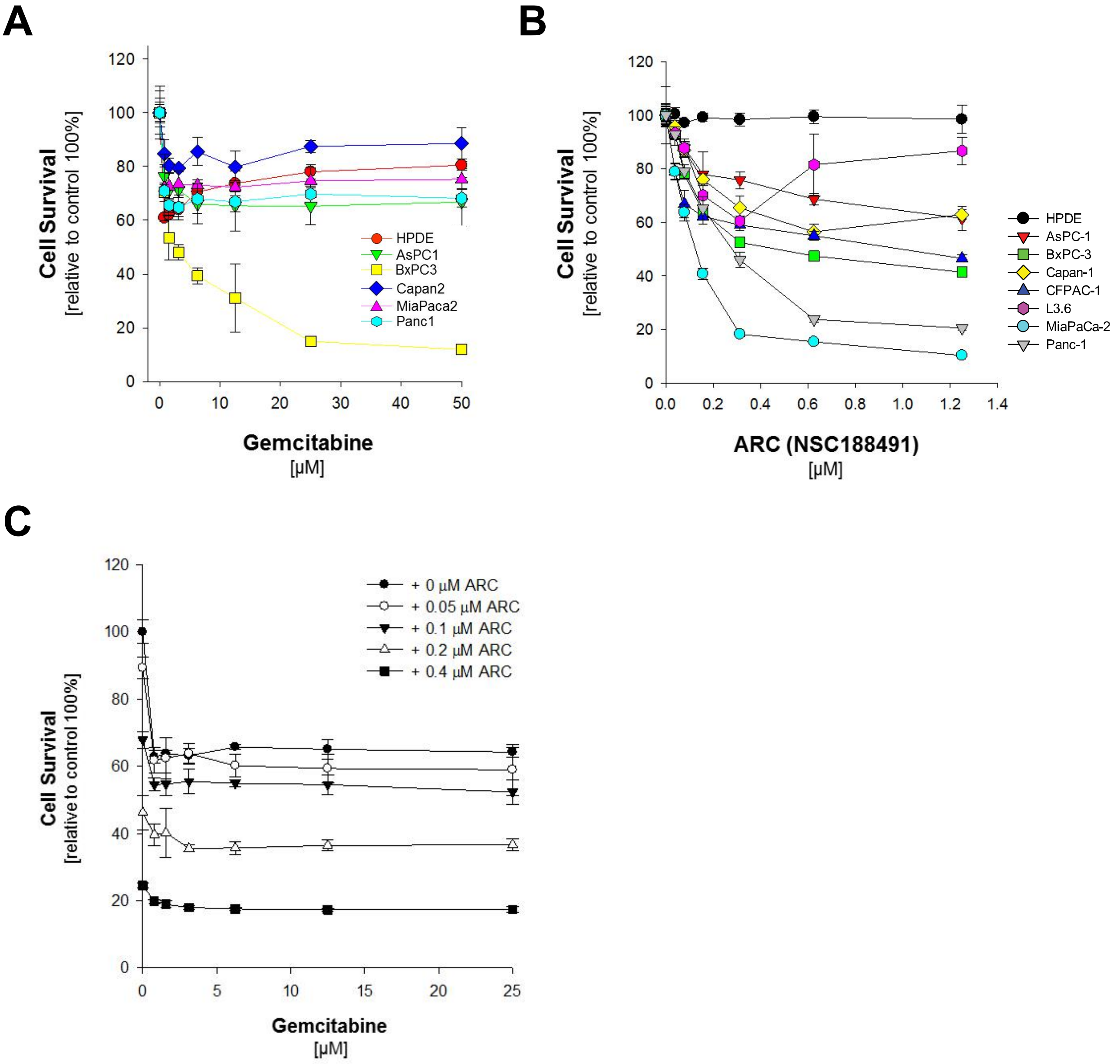

Supplemental Figure S1: A: Indicated cell lines were seeded in 96 well plates and treated with indicated concentrations of Gemcitabine for 48 hours. Cell survival was determined using MTT assay. **B:** Indicated cell lines were seeded in 96 well plates and treated with indicated concentrations of ARC (NSC188491) for 48 hours. Cell survival was determined using MTT assay. **C:** Panc1 cell were seeded in 96 well plates and treated with indicated concentrations of Gemcitabine in presence of 0, 0.05, 0.1, 0.2 or 0.4 M ARC (NSC188491) for 48 hours. Cell survival was determined using MTT assay.

<u>Supplemental Figure S2</u>

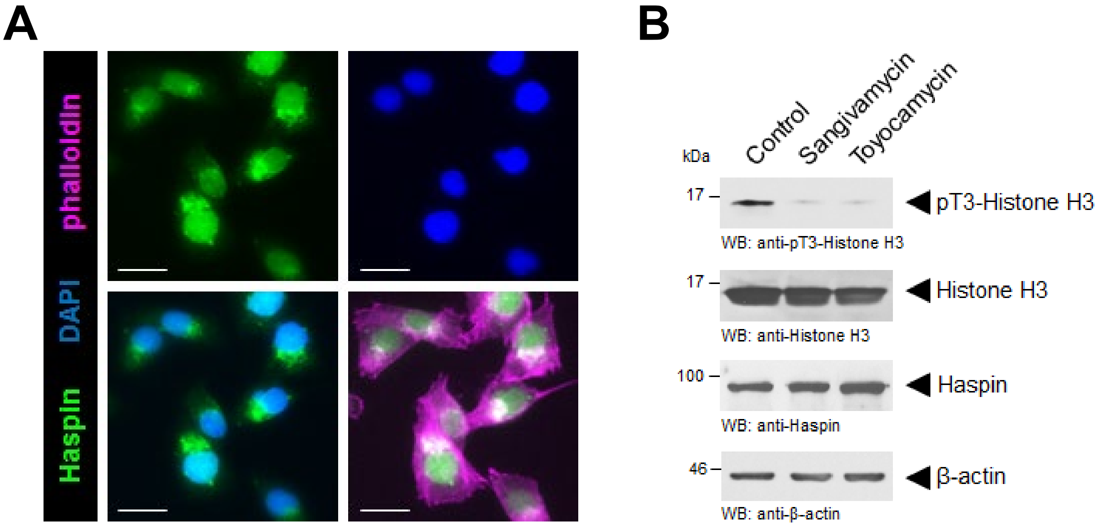

Supplemental Figure S2: A: MiaPaca2 cells were seeded on ibidi 8 well chambers, fixated and analyzed by immunofluorescence for expression and localization of Haspin. DAPI staining was included as a nuclear marker, and phalloidin staining as marker for cytoskeletal F-actin structures. The bar is 25 µm. **B:** MiaPaca2 cells were treated with Sangivamycin (500 nM), or Toyocamycin (500 nM) or control, for 16 hrs. Samples were resolved on SDS-PAGE and analyzed by Western blot for phosphorylation of Histone H3 at T3 (anti-pT3-Histone H3), total Histone H3 (anti-Histone H3), or Haspin (anti-Haspin). Western blotting for β-actin (anti-β-actin) served as control for equal loading.

Supplemental Figure S3

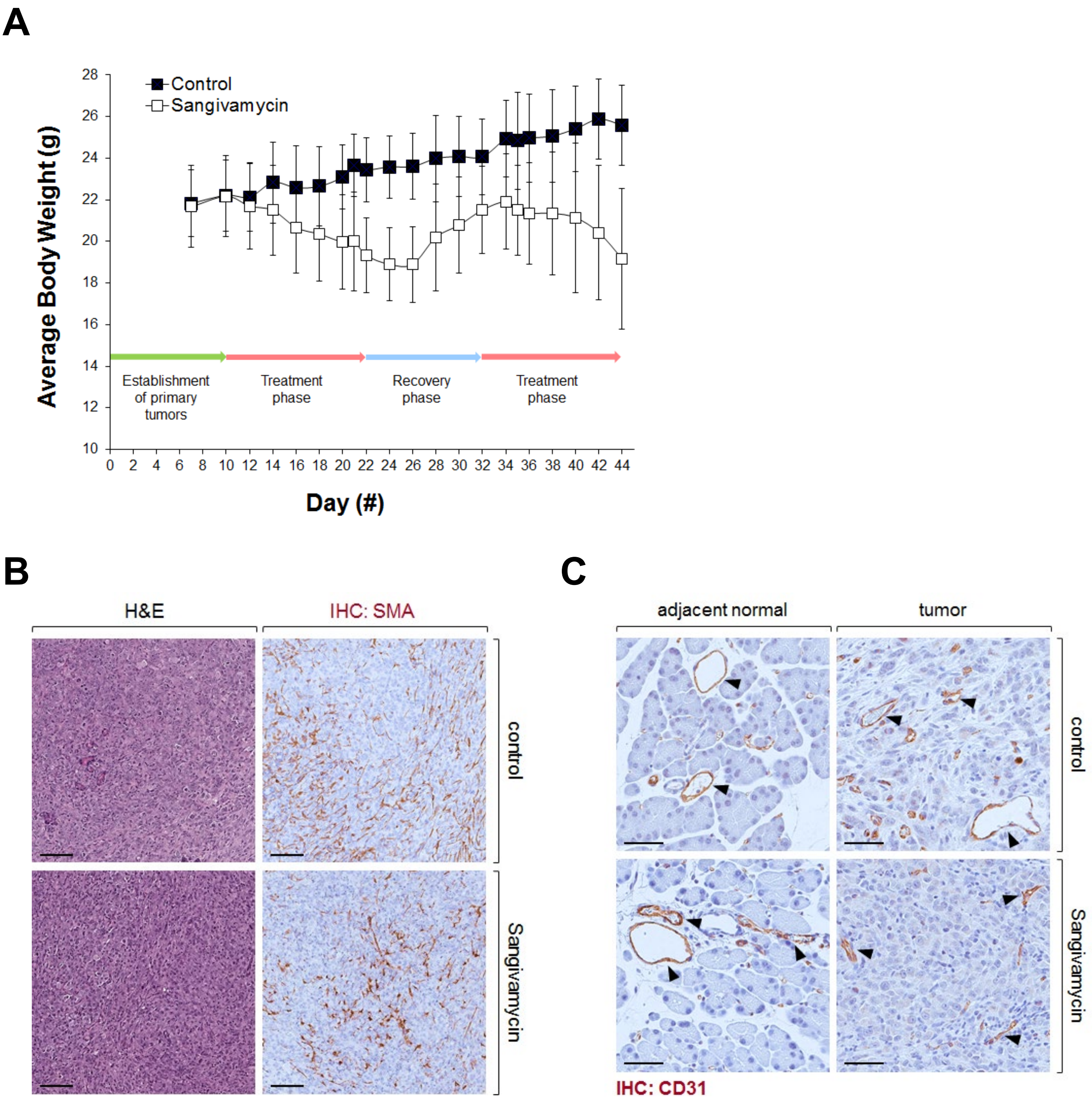

Supplemental Figure S3: A: Average body weight of mice during treatment**. B:**Orthotopic tumors from control or sangivamycin-treated mice. Shown is an H&E staining or IHC for smooth muscle actin (SMA). The bar is 100 μm. **C:** Adjacent normal and tumors from control or sangivamycin-treated mice with orthotopic tumors were analyzed for vasculature using IHC for CD31. The bar indicates 50 μm.

Supplemental Table 1

Antibody	Company/Source	Catalog Number	Species	IHC	IF/IF-IHC	WB	IP
CD31	Santa Cruz	sc-1506	goat	1:100			
cleaved Caspase 3	Cell Signaling	#9661	rabbit	1:600		1:1000	
cleaved PARP	Cell Signaling	#5625	rabbit			1:1000	
Cyclin B1 (V152)	Cell Signaling	#4135	mouse			1:2000	
DYRK1A	LSBioSciences	LS-C98731	rabbit	1:100		1:1000	
DYRK1A	Santa Cruz	sc-100376	rabbit			1:200	
DYRK2	Santa Cruz	sc-134324	rabbit			1:2000	
DYRK2	Sigma	HPA027230	rabbit	1:50		1:200	
Haspin	Bethyl	A302-241A	rabbit			1:1000	
Haspin	Novus	NBP1-26626	rabbit			1:2000	
Haspin	Sigma	HPA027422	rabbit	1:75	1:100		
Histone H3	Cell Signaling	#4499	rabbit			1:2000	
Histone H3	Abcam	ab1791	rabbit				5 µg/ml
Ki67	Abcam	ab15580	rabbit	1:500			
pS10-Histone H3	Abcam	ab5176	rabbit			1:1000	
pT3-Histone H3	Cell Signaling	#9714	rabbit	1:10 or 1:40		1:1000	
SMA	Abcam	ab5694	rabbit	1:200			
Survivin	Santa Cruz	sc-17779	mouse			1:250	2 µg/ml
YSK4	Novus	NBP1-83590	rabbit			1:200	
YSK4	MyBioSource	MBS8527095	rabbit	1:500			
β-actin	Sigma	A5441	mouse			1:5000	

Supplemental Table 1. **Antibodies and dilutions.** Antibodies used were from the following sources: Abcam (Cambridge, MA), Sigma (St. Louis, MO), Santa Cruz (Dallas, TX), LSBioSciences (Seattle, WA), Cell Signaling Technologies (Danvers, MA), Novus (Centennial, CO), or MyBioSource (San Diego, CA).
